# Supplementary material for: Climate Change Related Depression, Anxiety and Stress Symptoms Perceived by Medical Students
Source: Int J Environ Res Public Health. 2022 Jul 27;19(15):9142. doi: 10.3390/ijerph19159142 (PMC9332784; doi:10.3390/ijerph19159142)
Supplement: Supplementary file 1 [file ijerph-19-09142-s001.zip › ijerph-1781597-supplementary.pdf]

Table S1

Standardized loadings and pattern matrix of the PSQ-20-C for a two factor solution with oblique rotation.

Item[a]	*factor 1*	*factor 2*	h^{2b}	u^{2c}
You feel mentally exhausted	0.82	-0.09	0.63	0.37
You feel frustrated	0.80	0.03	0.66	0.34
Your problems seem to be piling up	0.79	0.01	0.63	0.37
You feel tense	0.78	0.02	0.62	0.38
You feel you're in a hurry	0.77	-0.01	0.60	0.40
You have many worries	0.75	0.10	0.61	0.39
You have trouble relaxing	0.71	-0.08	0.47	0.53
You fear you may not manage to attain your goals	0.67	0.04	0.47	0.53
You are afraid for the future	0.65	0.21	0.54	0.46
You have too many things to do	0.65	-0.03	0.41	0.59
You feel that too many demands are being made on you	0.62	0.00	0.39	0.61
You feel under pressure from deadlines	0.55	-0.12	0.29	0.71
You are full of energy	-0.02	0.73	0.53	0.47
You feel calm	0.17	0.72	0.62	0.38
You enjoy yourself	0.00	0.72	0.51	0.49
You feel rested	-0.04	0.65	0.41	0.59
You are lighthearted	0.13	0.63	0.46	0.54
You have enough time for yourself	-0.12	0.60	0.34	0.66
You feel you're doing things you really like	-0.27	0.58	0.33	0.67
You feel safe and protected	0.13	0.55	0.35	0.65

Annotation.[a] the present study used the validated German version of the PSQ-20. [b] communality, e.g. variance of the item explained for by the factors. [c] unexplained variance of the item.
